# *Aster spathulifolius* Maxim. Alleviates Primary Dysmenorrhea in a Mouse Model by Modulating Myometrial Contractions via NF-κB/COX-2 Pathway Inhibition

**DOI:** 10.1155/mi/1654087

**Published:** 2025-08-12

**Authors:** Min-Soo Kim, Kang-In Lee, Heung Joo Yuk, Yousang Jo, Hyungjun Kim, Ki-Sun Park

**Affiliations:** ^1^KM Convergence Research Division, Korea Institute of Oriental Medicine, Daejeon 34054, Republic of Korea; ^2^KM Science Research Division, Korea Institute of Oriental Medicine, Daejeon 34054, Republic of Korea

**Keywords:** *Aster spathulifolius* Maxim., NF-κB signaling, oxidative stress, primary dysmenorrhea, uterine contraction

## Abstract

Primary dysmenorrhea (PD) is characterized by excessive uterine contraction and ischemic vasoconstriction, primarily driven by elevated levels of prostaglandins (PGs; PGF2α) and inflammatory mediators. Nonsteroidal antiinflammatory drugs (NSAIDs) remain the standard treatment for PD; however, their associated adverse effects necessitate the use of alternative therapeutic strategies. *Aster spathulifolius* Maxim. is a perennial herb native to the coastal regions of Korea, that exhibits antiviral, anticancer, and antidiabetic effects. In this study, we investigated the potential of *Aster spathulifolius* Maxim. extract (PDR97) to alleviate PD in both animal models and human uterine smooth muscle cells (HUtSMCs). Our findings demonstrated that PDR97 significantly reduced pain-related responses and restored uterine morphology in PD-induced mice. Mechanistically, PDR97 suppressed the expression of uterine contraction-related proteins, decreased NF-κB phosphorylation, and downregulated the expression of proinflammatory cytokines. Furthermore, PDR97 effectively inhibited PGF2α- and interleukin-1β (IL-1β)-induced reactive oxygen species (ROS) production in both the PD mouse model and HUtSMCs, exhibiting potent antioxidant properties. Notably, PDR97 modulated NF-κB signaling—a key regulatory pathway associated with uterine contraction and pain relief—and its antioxidant effects contributed to the suppression of inflammatory and oxidative stress-mediated signaling. Collectively, these findings highlight the potential of PDR97 as a promising natural therapeutic agent for PD, with potential applications in other gynecological disorders associated with inflammation and oxidative stress.

## 1. Introduction

Dysmenorrhea is classified into two types: primary dysmenorrhea (PD) and secondary dysmenorrhea [1]. PD occurs without any underlying gynecological pathology, whereas secondary dysmenorrhea is associated with conditions like endometriosis and uterine leiomyoma [2]. Although PD is not caused by anatomical abnormalities or pathological conditions, it is characterized by severe cramping pain in the lower abdomen that occurs just before or during menstruation and lasts for ~24–72 h. Moreover, PD is often accompanied by systemic symptoms such as nausea, vomiting, fatigue, headache, and diarrhea. Due to its considerable impact on daily activities, including academic and professional performance, PD is considered a major gynecological disorder that reduces quality of life [3, 4].

The pathophysiology of PD is primarily attributed to the excessive production of prostaglandins (PGs) and leukotrienes (LTs) in the endometrium [5]. Among these, PGF2α plays a crucial role in stimulating uterine smooth muscle contraction and vasoconstriction, which leads to ischemic pain. Because the endometrial lining sheds during menstruation, increased levels of PGF2α and PGE2 can trigger strong uterine contractions and reduce uterine blood flow, thereby activating nociceptors and amplifying pain perception [6]. Moreover, LTs and vasopressin further exacerbate menstrual pain. Clinical studies have demonstrated that women with higher PGF2α concentrations tend to experience more severe and frequent dysmenorrhea. Consequently, therapeutic strategies that modulate PGF2α activity and promote uterine smooth muscle relaxation have been proposed as potential interventions for the management of PD.

Nonsteroidal antiinflammatory drugs (NSAIDs) are the first-line of treatment for PD. The primary mechanism of action of NSAIDs involves the inhibition of cyclooxygenase (COX) enzymes, thereby suppressing PG synthesis. By targeting COX-2, NSAIDs effectively reduce PGF2α and PGE2 levels, alleviating uterine contractions and inflammation [7, 8]. However, NSAIDs also inhibit COX-1, which plays a crucial role in maintaining gastric mucosal integrity and renal function. This nonselective inhibition results in adverse effects such as gastrointestinal irritation, renal impairment, and increased cardiovascular risk. Moreover, prolonged NSAID use may lead to tolerance, necessitating higher doses or additional analgesics for pain relief [9]. These limitations highlight the urgent need for safe and effective alternative therapeutic strategies.

Recent studies have focused on developing natural product-based treatments as potential alternatives to NSAIDs [10]. Natural compounds have been shown to regulate PG synthesis, exert antiinflammatory effects, and promote uterine relaxation. For instance, curcumin, derived from turmeric, inhibits COX-2 activity and reduces PGE2 production [11]. Ginger extract exhibits NSAID-like antiinflammatory properties by modulating PG and LT production [12]. Glycyrrhizin, a bioactive component of *Glycyrrhiza glabra* (licorice), exerts antiinflammatory effects by stabilizing the uterine smooth muscle activity [13]. Given these promising findings, further research into the mechanism of action of natural products is essential to validate their clinical efficacy and establish novel and safer treatment options for PD.

*Aster spathulifolius* Maxim., a perennial herb belonging to the Asteraceae family, is native to the coastal regions of Japan and Korea [14]. The extracts of *Aster spathulifolius* Maxim. leaves (PDR97) have demonstrated diverse bioactive properties, including antiviral, and antidiabetic effects [15, 16]. However, its precise biochemical mechanisms and therapeutic potential in alleviating PD remain largely unexplored. In this study, we investigated the efficacy of PDR97 in alleviating PD using an animal model that mimics PD-related pain and examined its biochemical mechanisms in human uterine smooth muscle cells (HUtSMCs). The findings of this study provide insight into the potential application of PDR97 as a natural therapeutic agent or dietary supplement for PD, offering an alternative to NSAIDs with improved safety and efficacy.

## 2. Materials and Methods

### 2.1. Chemicals and Reagents

Estradiol benzoate, ibuprofen, and oxytocin were purchased from Sigma–Aldrich (St. Louis, MO, USA). The primary antibodies used against the oxytocin receptor (Cat# AVR-013) were purchased from Alomone Labs (Jerusalem BioPark, Jerusalem, Israel). COX-2 (Cat# SC-1745) and tumor necrosis factor α (TNF-α) (Cat# SC-52746) were purchased from Santa Cruz Biotechnology (Dallas, Texas, USA). β-actin (Cat# 4967 S), p44/42 mitogen-activated protein kinases (ERK1/2) (Cat# 9102 S), p-ERK1/2 (Cat# 9101 S), p-myosin light chain 2 (p-MLC20) (Cat# 3675 S), and p-nuclear factor ĸB (p-NF-ĸB) (Cat# 3033 S), NF-ĸB (Cat# 8482 S), and Lamin A/C (Cat# 4777 S) were obtained from Cell Signaling Technology (Danvers, Massachusetts, USA). MLC-20 (Cat# ab48003) and inducible nitric oxide synthase (iNOS) (Cat# ab15323) were purchased from Abcam (Cambridge, MA, USA). Interleukin-6 (IL-6) (Cat# P620), Alexa Fluor 488 donkey anti-rabbit IgG (Cat# A21206), 4,6-diamidino-2-phenylindole (DAPI) nuclear staining dye (Cat# D1306), and dihydroethidium (DHE) (Cat# D1168) were obtained from Invitrogen (Carlsbad, California, USA). PGF2α was purchased from Cayman Chemical (Cat# 16010, Ann Arbor, MI, USA) and IL-1b was purchased from Sigma (Cat# SRP3083, St. Louis, MO, USA).

### 2.2. PDR97 Sample Preparation

*Aster spathulifolius* Maxim. was purchased three times between May and August 2022 from Milal Farm (Hwaseong-si, Gyeonggi-do, Republic of Korea). The plant material was identified and authenticated based on morphological characteristics. Only the leaves were used, after manually removing the stems. The leaves were thoroughly rinsed with running tap water to remove dust and surface impurities, then shade-dried for 5–7 days in a well-ventilated area at ambient temperature (~25°C, relative humidity ~ 50%). The dried leaves were processed by a professional natural product extraction company (KOC Biotech, Yuseong-Gu, Daejeon, Republic of Korea). For extraction, 100 g of dried leaves was macerated in 15 L of 70% ethanol (ethanol:distilled water = 7:3, v/v) at room temperature (25 ± 2°C) for 7 days, protected from light. The extract was filtered through a 100-mesh sieve and concentrated under reduced pressure at 40°C using a rotary evaporator. The concentrated extract was then freeze-dried at−55°C under vacuum (0.05 mbar) for 48 h to obtain a powdered form of the 70% ethanol extract of *Aster spathulifolius* Maxim. leaves (referred to as PDR97). All freeze-dried samples were stored in airtight containers at −80°C in an ultra-low temperature freezer at the Korea Institute of Oriental Medicine (KIOM), until further use.

### 2.3. Experimental Animals and In Vivo Experimental Design

All experimental procedures were performed in accordance with the protocols approved by the Animal Care and Use Committee of the KIOM (approval number: 22-067) and complied with the guidelines of the National Institutes of Health and Animal Research: Reporting of In Vivo Experiments (ARRIVE). Every effort was made to minimize the number of animals used and their suffering.

**Figure 1:**
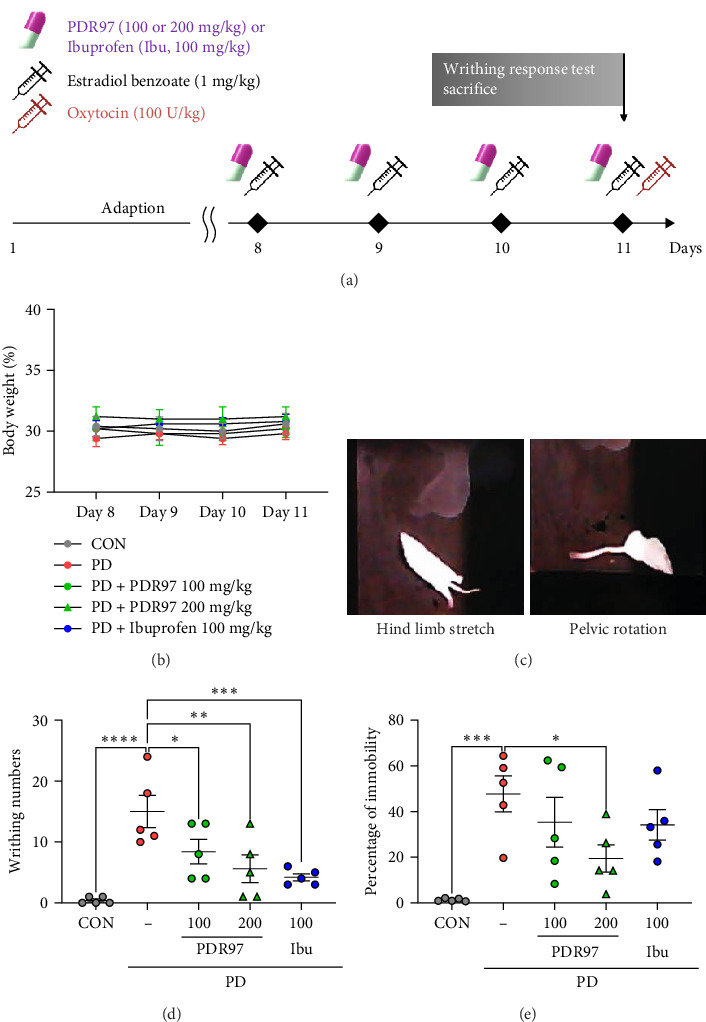
Study design and effect of PDR97 in oxytocin-induced writhing response test. (A) Schematic diagram of the experimental design. After a 7-day adaption period, mice were administered with PDR97 (100 or 200 mg/kg, p.o.) or ibuprofen (100 mg/kg, p.o.) for 4 days, followed 30 min later by an intraperitoneal injection of estradiol benzoate (1 mg/kg, i.p.) or saline (i.p.). On the 11th day, mice were administered oxytocin (100 U/kg, i.p.) and subsequently underwent a writhing response test. After the experiment, the mice were euthanized and their uteri were collected for further analysis. (B) Body weight of mice during treatment period. (C) Representative writhing responses including stretching of the hind limbs and rotation of the pelvis. (D, E) Writhing time (D) and percentage of time spent immobile (E) were measured for a 30 min period following the administration of oxytocin. Data are expressed as mean ± SEM. Statistical analysis was conducted using one-way ANOVA, followed by Fisher's LSD test. *⁣*^*∗*^*p*  < 0.05, *⁣*^*∗∗*^*p*  < 0.01, *⁣*^*∗∗∗*^*p*  < 0.001, *⁣*^*∗∗∗∗*^*p*  < 0.0001.

Seven-week-old female ICR mice were obtained from Orient Bio (Seongnam, Gyeonggi, Republic of Korea) and allowed to acclimatize for 1 week. All mice were housed in a pathogen-free facility with 12 h light/12 h dark cycles (condition: 22 ± 2°C temperature; 50% ± 10% humidity) and unrestricted access to normal water and food. The mice were randomly divided into five groups with five mice in each group, as follows: normal control group (CON), PD group, low-dose PDR97 + PD group (PD + PDR97 100 mg/kg), high-dose PDR97 + PD group (PD + PDR97 200 mg/kg), and ibuprofen + PD group (PD + ibuprofen 100 mg/kg). Ibuprofen was used as the positive control for comparison with PDR97 [17]. PDR97 or ibuprofen was dissolved in saline and administered orally for 4 days, while mice in the CON and PD groups received saline for the same period of time. Following gavage, an intraperitoneal injection of estradiol benzoate (1 mg/kg) was administered to mice, with the exception of those in the CON group, who received a saline injection. On day 4, oxytocin (100 U/kg) was administered via an intraperitoneal injection 30 min after the last treatment to induce dysmenorrhea [18, 19]. The mice were weighed daily throughout the treatment period. A schematic overview of the experimental protocol is shown in Figure 1A.

### 2.4. Oxytocin-Induced Writhing Response Test

To observe the pain response to oxytocin-induced dysmenorrhea, behavioral tests were performed as previously described [20, 21]. Following oxytocin injection, the mice were placed in an open field arena (40 cm × 40 cm × 40 cm) and their writhing response was observed for 30 min. The writhing responses primarily comprised of pelvic rotation and contraction of the abdomen, accompanied by stretching of the hind limbs. The number of writhes and duration of immobility was recorded and subsequently assessed using a video tracking system (HVS Image, Bicester, UK).

### 2.5. Tissue Sample Preparation

All mice were sacrificed by cervical dislocation after behavioral testing. The uterine tissues were isolated, and the weight and length of the uterine horn were measured. Half of the tissue samples were stored at −80°C until use for immunoblotting, while the remaining samples were fixed in 4% paraformaldehyde for histological analysis. Blood samples were collected and subsequently centrifuged at 1500 × *g* for 10 min at 4°C, to produce serum for further analysis using flow cytometry.

### 2.6. Histological Examination

The fixed tissue samples were embedded in paraffin and cut into multiple 4 μm-thick sections for hematoxylin–eosin staining [18]. Morphological examinations of the slices were performed using a light microscope. Stained images were captured with an equipped camera, and the thicknesses of the myometrium and basalis layers of the endometrium were measured using ImageJ software (version 1.52, Bethesda, MD, USA).

### 2.7. Immunoblotting

The uterine tissue was homogenized in cold lysis buffer with a protease inhibitor cocktail (GenDEPOT, Texas, USA), and the homogenate was centrifuged at 14,000 × *g* for 30 min. Equivalent amounts of protein were loaded onto 4%–20% TGX gels (Bio-Rad, Hercules, CA, USA) at 100 V for 100 min and then transferred to the polyvinylidene difluoride membranes. The membranes were blocked with 5% skim milk solution at 25°C for 1 h, and subsequently probed with specific primary antibodies at 4°C overnight. After washing with tris-buffered saline containing Tween-20 (TBST), the membranes were immersed in the corresponding secondary antibodies and visualized using a digital imaging system (Bio-Rad). Protein density was quantified using Image Gauge software (Fujifilm, Tokyo, Japan). Details about the primary and secondary antibodies used is provided in Table S1.

### 2.8. Immunofluorescence Staining

To measure p-NF-κB expression in the uterine tissue, immunofluorescence staining was performed. Paraffin-embedded 4 μm-thick sections were deparaffinized and hydrated. The sections were then permeabilized with 0.15% Triton X-100 in phosphate buffered saline (PBST) and subsequently blocked via incubation with 0.2% bovine serum albumin in PBST containing 5% horse serum (Vector Laboratories, California, USA) at 25°C for 1 h. To induce NF-κB activation in the uterine tissue, PGF2α (10 μM) and IL-1β (10 ng/mL) were used as inflammatory stimuli, as described in previous study [22]. Thereafter, the sections were incubated overnight with p-NF-κB antibody at 4°C. After washing with PBST, the sections were incubated with Alexa Fluor 488-conjugated anti-rabbit secondary antibody. To counterstain the nuclei, DAPI solution (Cat# D9542, Sigma–Aldrich) was used. Stained images were acquired using a fluorescence microscope (BP73, Olympus, Seoul, South Korea), and fluorescence intensity was analyzed using ImageJ software.

### 2.9. DHE Staining

Reactive oxygen species (ROS) levels were measured in situ using the fluorescent probe DHE. The 4 μm-thick tissue sections were exposed to DHE (10 μM in PBS) in a dark environment for 30 min at 37°C. Following washing with PBS, the sections were subjected to counterstaining with DAPI to facilitate the identification of nuclei. Images were captured using a fluorescence microscope and positive areas were subsequently analyzed using the ImageJ software.

### 2.10. Cell Culture

HUtSMCs were purchased from PromoCell (Cat# C-12575, PromoCell, Heidelberg, Germany). Smooth muscle cell growth medium-2 supplemented with the provided supplement set (Cat # C-22062, PromoCell, Heidelberg, Germany) was used as the culture medium. The cell culture conditions were maintained according to the manufacturer's protocol.

### 2.11. Measurement of ROS Levels

Intracellular ROS levels were measured in HUtSMCs using 2,7-dichlorodihydrofluorescein diacetate (DCFDA; Cat# D399, Invitrogen, MA, USA) [23]. Cells were divided into four experimental groups: untreated and PBS only cells (negative control), PGF2α (10 µM) + IL-1β (10 ng/mL)-treated cells (positive control for ROS induction), PGF2α + IL-1β + N-acetylcysteine (NAC, 5 mM) (antioxidant control), and PGF2α + IL-1β + PDR97 (test group). After treatment, cells were incubated with 10 µM DCFDA in serum-free medium for 30 min at 37°C in dark, followed by washing with PBS. ROS levels were then quantified using a microplate reader (excitation: 485 nm and emission: 535 nm) and a flow cytometer (BD LSRFortessa X-20; Becton-Dickinson, San Jose, CA, USA).

### 2.12. Luciferase Activity Assay

The NF-κB-Luc promoter vector plasmid was transiently transfected into HUtSMCs using Lipofectamine 3000 (Cat# L3000-015, Invitrogen, MA, USA). The assay was performed using the Dual-Glo Luciferase Reporter Assay System (Cat# E2920, Promega, WI, USA) and luminescence was measured using the Centro XS³ LB 960 Microplate Reader (Berthold, Oak Ridge, TN, USA).

### 2.13. NF-*κ*B Translocation Assay

To assess NF-κB translocation in HUtSMCs, immunostaining was performed using a confocal-specific dish (Cat# CLS354118, Sigma, St. Louis, MO, USA). When the cells reached nearly 100% confluence, the medium was replaced with serum-free medium for 12 h, followed by pretreatment with PDR97 for 30 min. Subsequently, PGF2α (10 μM) and IL-1β (10 ng/mL) were administered for 30 min. Cells were then fixed with 4% paraformaldehyde, permeabilized with 0.1% Triton X-100 for 10 min. The primary NF-κB antibody was diluted 1:100 in DAKO diluent solution and incubated at 4°C for 24 h. The secondary antibody was diluted 1:1000 in DAKO diluent solution and incubated at 4°C for 1 h. Fluorescence microscopy was used to observe cells. For cytoplasmic and nuclear protein fractionation of HUtSMCs, a fractionation kit (Cat# K266−100, Biovision, Milpitas, CA, USA) was used according to the manufacturer's instructions. Immunoblotting was performed as described in Section 2.7.

### 2.14. Statistical Analyses

Statistical analyses were performed using GraphPad Prism 9.0 (San Diego, CA, USA). Data are expressed as mean ± standard error of mean (SEM). Levene's test was performed to test for homogeneity of variance. One-way analysis of variance (ANOVA), followed by Fisher's least significant difference test was performed for multiple group comparisons. Statistical significance was set at *p*  < 0.05.

## 3. Results

### 3.1. PDR97 Ameliorates Pain-Related Responses in Mice With PD

After a 1-week adaptation period, the mice received the drug orally for 4 days, followed by intraperitoneal injection of estradiol benzoate (Figure 1A). The mice were weighed daily throughout the treatment period, and no significant differences were observed between the groups (Figure 1B). To study the pain behavior of mice with PD, oxytocin was administered intraperitoneally on the last day. Oxytocin induced immediate writhing reactions consisted mainly of abdominal wall contraction, pelvic rotation, and hindlimb extension (Figure 1C). The CON group showed almost no writhing behavior, whereas the PD group showed an average of 15 writhing behaviors, indicating that the PD model was effectively established. However, the administration of PDR97 significantly reduced the frequency of writhing behavior (*p*  < 0.05, PDR97 100 mg/kg; *p*  < 0.01, PDR97 200 mg/kg; Figure 1D). Ibuprofen, a positive control for the treatment of PD, also resulted in a decrease in writhing numbers compared to the CON group (*p*  < 0.001). Furthermore, the PD model showed a substantial increase in oxytocin-induced immobility, which was significantly ameliorated by PDR97 200 mg/kg treatment (*p*  < 0.05; Figure 1E). These results suggest that PDR97 mitigates the severity of pain-related responses in mice with PD.

### 3.2. PDR97 Restores Uterine Morphology in Mice With PD

**Figure 2:**
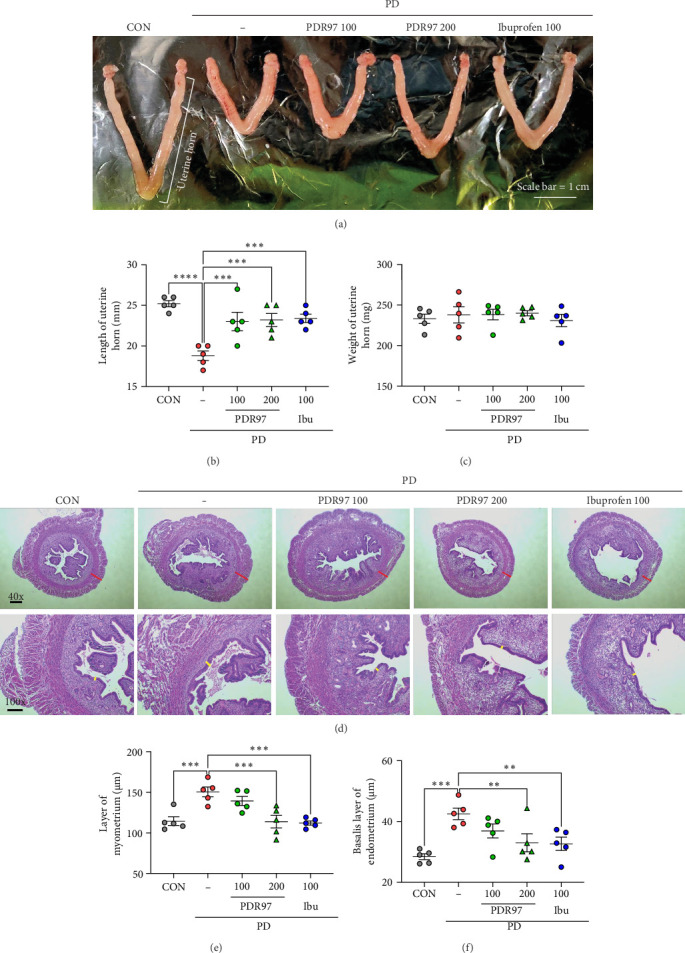
Effects of PDR97 on uterine morphology of mice with PD. (A) Representative images of uteri of each group (scale bar: 1 cm). (B, C) Length from ovary to cervix (B) and weight of the uterine tissue (C) were measured. (D) Representative histological images of hematoxylin–eosin-stained mouse uterine sections (upper: magnification 40x, scale bar: 50 μm; lower: magnification 100x, scale bar: 100 μm). Red line indicates the layer of myometrium; yellow line indicates the basalis layer of endometrium. (E, F) Thickness of the layer of myometrium (E) and the basalis layer of endometrium (F) were measured. Data are expressed as mean ± SEM. Statistical analysis was conducted using one-way ANOVA, followed by Fisher's LSD test. *⁣*^*∗∗*^*p*  < 0.01, *⁣*^*∗∗∗*^*p*  < 0.001, *⁣*^*∗∗∗∗*^*p*  < 0.0001.

Gross examination was performed to observe morphological changes in the uterus. Compared to the CON group, the uteri of mice in the PD group showed an excessively contracted shape (Figure 2A). Measurement of the uterine horn length revealed that the PD group exhibited a reduced length compared to the CON group (*p*  < 0.0001). Interestingly, the administration of PDR97 and ibuprofen restored the uterine length to normal levels (*p*  < 0.001; Figure 2B). However, no significant differences in uterine weight were observed between groups (Figure 2C). Hematoxylin–eosin staining was performed to investigate potential histological alterations in the uterine sections. The PD group exhibited an atypical structure characterized by an increased length of the myometrial layer as well as the basalis layer of the endometrium (Figure 2D). However, these alterations were notably restored by both PDR97 200 mg/kg and ibuprofen treatment (*p*  < 0.001 for the myometrium layer, Figure 2E; *p*  < 0.01 for the basalis layer of the endometrium; Figure 2F). These results suggested that PDR97 could modulate PD-induced morphological changes in the uterus.

### 3.3. PDR97 Decreases Expression of Uterine Contraction-Related Proteins in Mice With PD

**Figure 3:**
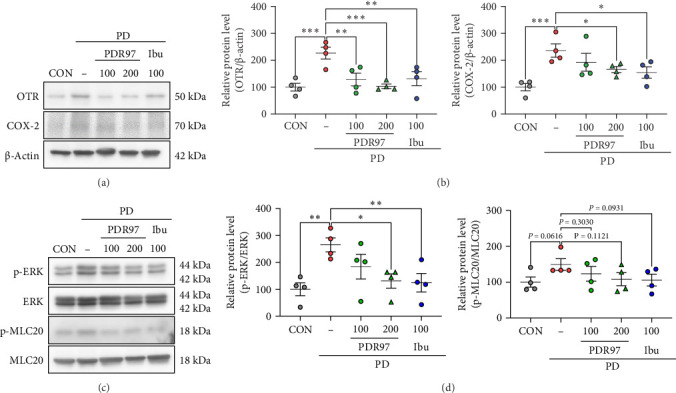
Effects of PDR97 on uterine contraction-related protein expression in mice with PD. (A) Immunoblots detecting OTR and COX-2. (B) Expression levels of OTR and COX-2, normalized to β-actin, are presented as histograms. (C) Immunoblots detecting ERK, MLC20, phosphorylated ERK, and phosphorylated MLC20. (D) Expression levels of p-ERK and p-MLC20, normalized to total ERK and total MLC20 respectively, are presented as histograms. The relative expression level of protein was designated as 100% in the CON group. Data are expressed as mean ± SEM. Statistical analysis was conducted using one-way ANOVA, followed by Fisher's LSD test. *⁣*^*∗*^*p*  < 0.05, *⁣*^*∗∗*^*p*  < 0.01, *⁣*^*∗∗∗*^*p*  < 0.001.

Given that OTR binds to oxytocin to cause abnormal contraction of the uterine smooth muscle and is thought to be a key factor in causing menstrual pain along with COX-2 [18], we investigated the effect of PDR97 on the expression levels of OTR and COX-2 in PD mice (Figure 3A). We found that the expression of OTR was significantly increased in the uterus of mice in the PD group compared to that in the CON group (*p*  < 0.001), whereas the PDR97 group showed a dose-dependent decrease in the levels of OTR (*p*  < 0.01, PDR97 100 mg/kg; *p*  < 0.001, PDR97 200 mg/kg). Similarly, the elevated expression of COX-2 observed in the PD group (*p*  < 0.001) was notably reduced in the 200 mg/kg PDR97 group (*p*  < 0.05; Figure 3B).

ERK is a pivotal component of the OTR-induced signaling pathway, and its activation increases uterine contractions by promoting the phosphorylation of MLC20 [24]. Therefore, we assessed the effects of PDR97 on phosphorylated ERK and MLC20 levels (Figure 3C). We found an increase in phosphorylated ERK in the PD group compared to that in the CON group (*p*  < 0.01), which was restored by PDR97 administration at a dose of 200 mg/kg (*p*  < 0.05; Figure 3D). MLC20 phosphorylation tended to be comparable to ERK phosphorylation, although this was not statistically significant. Ibuprofen administration showed effects similar to those of PDR97. Collectively, these results suggest that PDR97 exerts a positive effect on PD by modulating the expression of proteins associated with uterine contractions.

### 3.4. PDR97 Decreases the Phosphorylation Levels of NF-ĸB and Proinflammatory Cytokines in Mice With PD

**Figure 4:**
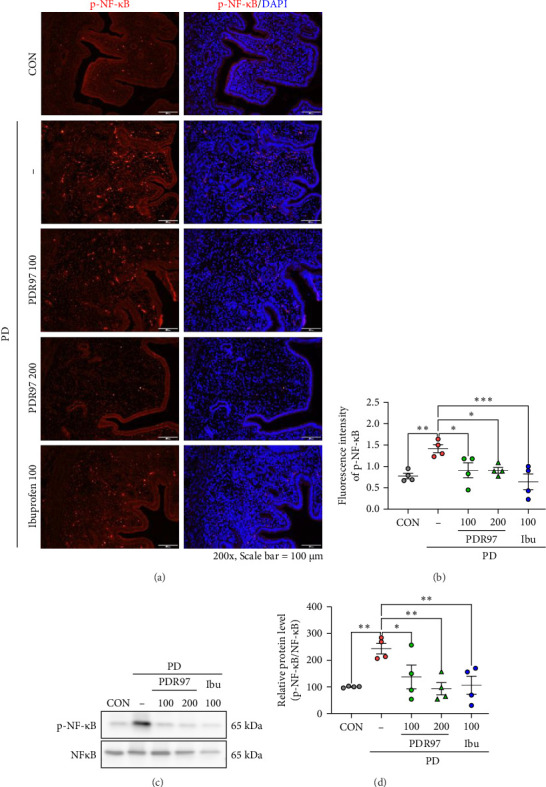
Effects of PDR97 on NF-ĸB protein phosphorylation in the uterus of mice with PD. (A) Representative immunofluorescence images taken at 200x original magnification (scale bar = 100 μm), stained for p-NF-ĸB (red). DAPI (blue) was used as a counterstain for nuclear staining. (B) Quantification of fluorescence intensity of p-NF-ĸB in the uterus. At least three uterine sections from each mouse were used for staining. (C) Immunoblots detecting NF-ĸB and p-NF-ĸB. (D) Expression levels of p-NF-ĸB, normalized to total NF-ĸB, are presented as histograms. The relative expression level of protein was designated as 100% in the CON group. Data are expressed as mean ± SEM. Statistical analysis was conducted using one-way ANOVA, followed by Fisher's LSD test. *⁣*^*∗*^*p*  < 0.05, *⁣*^*∗∗*^*p*  < 0.01, *⁣*^*∗∗∗*^*p*  < 0.001.

As it is known that NF-ĸB is a key upstream regulator of COX-2 [25], we next investigated whether PDR97 affects NF-ĸB phosphorylation in the uterus. Compared with CON group, fluorescence intensity associated with p-NF-ĸB in the PD group was significantly increased (*p*  < 0.01). However, administration of PDR97 notably reversed this change in a dose-dependent manner (*p*  < 0.05; Figure 4A,B), with an effect similar to that of ibuprofen. In accordance with the findings of immunofluorescence staining, administration of PDR97 resulted in the normalization of p-NF-ĸB expression, induced by PD, as evidenced by immunoblotting analysis in the uterus (*p*  < 0.01 for PD, *p*  < 0.05 for PDR97 at 100 mg/kg, *p*  < 0.01 for PDR97 at 200 mg/kg; Figure 4C,D).

**Figure 5:**
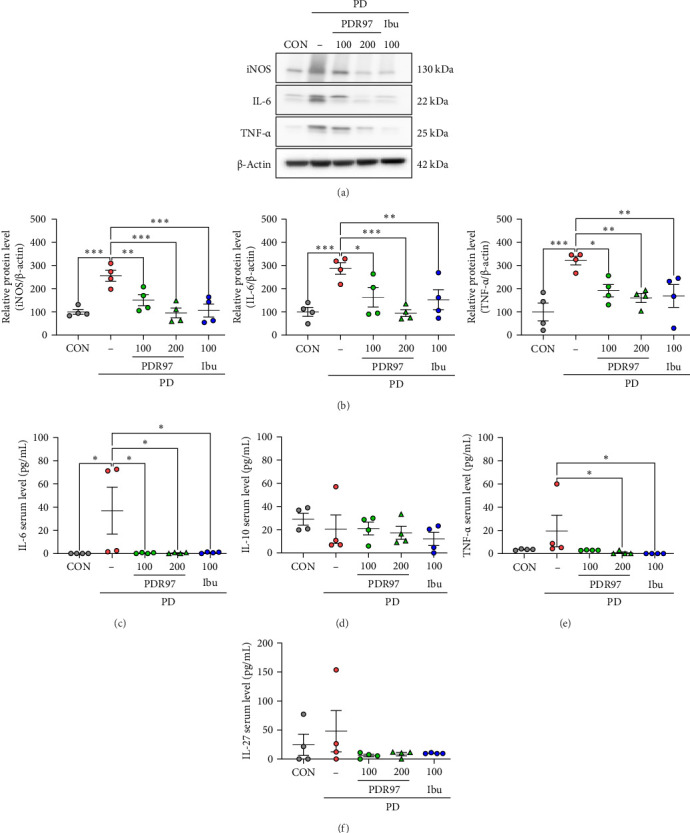
Effects of PDR97 on inflammatory mediators in the uterus of mice with PD. (A) Immunoblots detecting iNOS, IL-6, and TNF-α. (B) Expression levels of iNOS, IL-6, and TNF-α, normalized to β-actin, are presented as histograms. The relative expression level of protein was designated as 100% in the CON group. (C–F) Serum concentrations of inflammatory mediators, including IL-6 (C), IL-10 (D), TNF-α (E), and IL-27 (F). Data are expressed as mean ± SEM. Statistical analysis was conducted using one-way ANOVA, followed by Fisher's LSD test. *⁣*^*∗*^*p*  < 0.05, *⁣*^*∗∗*^*p*  < 0.01, *⁣*^*∗∗∗*^*p*  < 0.001.

Given the established role of NF-ĸB in the induction of proinflammatory gene expression, subsequent analyses focused on the expression of inflammatory cytokines. As shown in Figure 5A,B, there was a significant increase in the expression levels of iNOS, IL-6, and TNF-α in the uterus of the PD group in comparison with the CON group (*p*  < 0.001); however, this upregulation was alleviated markedly by PDR97 administration (iNOS: *p*  < 0.01 for PDR97 at 100 mg/kg, *p*  < 0.001 for PDR97 at 200 mg/kg; IL-6: *p*  < 0.05 for PDR97 at 100 mg/kg, *p*  < 0.001 for PDR97 at 200 mg/kg; TNF-α: *p*  < 0.05 for PDR97 at 100 mg/kg, *p*  < 0.01 for PDR97 at 200 mg/kg). Treatment with ibuprofen exhibited effects analogous to those of the PDR97 treatment (Figure 5B). The concentration of inflammatory cytokines in the serum was measured using FACS analysis. The concentrations of IL-6 and TNF-α increased in the PD group (*p*  < 0.05 for IL-6; *p*=0.06 for TNF-α; Figure 5C,E) and reduced in the PDR97 treatment group (IL-6: *p*  < 0.05 for PDR97 at 100 and 200 mg/kg; TNF-α: *p*  < 0.05 for PDR97 at 200 mg/kg), but there was no significant difference in the concentrations of IL-10 and IL-27 (Figure 5D,F). These results suggest that PDR97 may play a role in the antidysmenorrhea activity through the inhibition of NF-ĸB and downstream proinflammatory cytokines.

### 3.5. PDR97 Inhibits ROS Production Induced by PGF2α and IL-1β in Mouse Model and Human Uterine Smooth Muscle Cells

**Figure 6:**
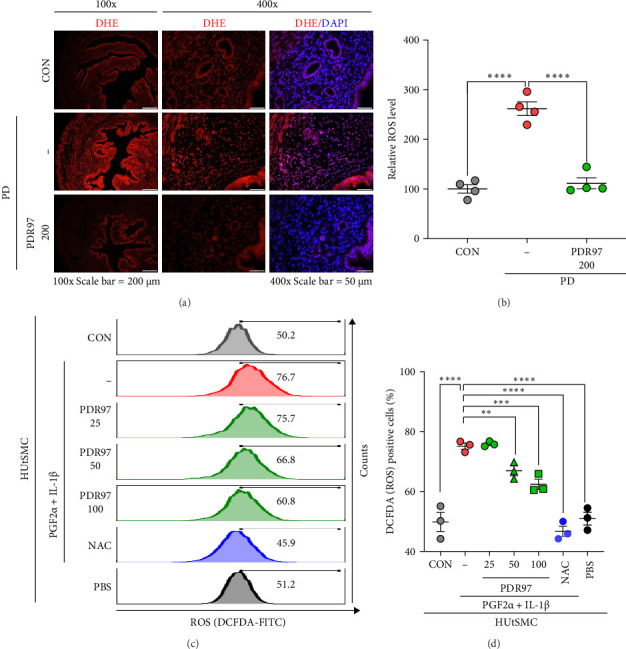
Effects of PDR97 on ROS production in vivo and in vitro. (A) Representative images taken at 100x (scale bar = 200 μm) and 400x (scale bar = 50 μm) original magnification stained with DHE (red) to detect ROS. DAPI (blue) was used as a counterstain for nuclear staining. (B) Quantification of DHE fluorescence in the uterus. At least three uterine sections from each mouse were used for staining. (C) Analysis of intracellular ROS levels using flow cytometry. (D) Number of positive cells for DCFDA fluorescence in (C). Data are expressed as mean ± SEM. Statistical analysis was conducted using one-way ANOVA, followed by Fisher's LSD test. *⁣*^*∗*^*p*  < 0.05, *⁣*^*∗∗*^*p*  < 0.01, *⁣*^*∗∗∗∗*^*p*  < 0.0001.

We further examined the expression of ROS in the uterine tissue by DHE staining, given the established correlation between proinflammatory cytokines and increased ROS production (Figure 6A). As expected, a significant increase in ROS levels was observed in the uteri of the PD group compared to that in the CON group (*p*  < 0.0001). However, PDR97 treatment markedly inhibited ROS production (*p*  < 0.0001; Figure 6B). The uterotonic hormone PGF2α and the proinflammatory cytokine IL-1β have been reported to induce ROS production in uterine muscle cells. To investigate whether PDR97 attenuates ROS generation, we measured ROS levels in HUtSMCs treated with PGF2α and IL-1β. Our results demonstrated a significant increase in ROS production upon PGF2α and IL-1β stimulation. However, PDR97 effectively reduced ROS levels in a dose-dependent manner (*p*  < 0.05 at 50 μg/mL; *p*  < 0.01 at 100 μg/mL; Figure 6C,D). This finding aligns with the results from in vivo models, suggesting that PDR97 may exert a direct protective effect on uterine smooth muscle cells.

### 3.6. PDR97 Inhibits NF-*κ*B Activation—a Key Mechanism Associated With Uterine Contraction and Pain Relief

**Figure 7:**
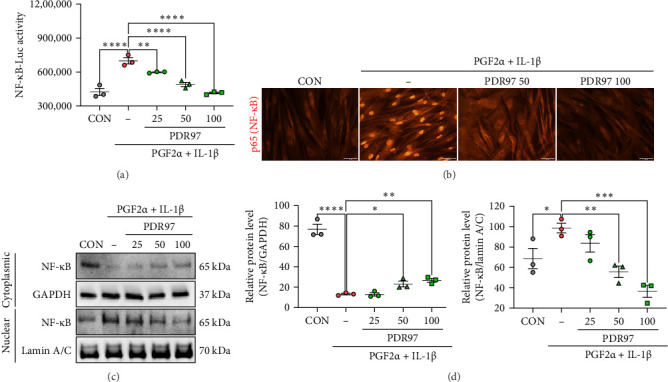
Inhibitory effect of PDR97 on NF-κB activation in HUtSMCs. (A) NF-κB luciferase activity. HUtSMCs were pretreated with PDR97 for 30 min, followed by coincubation with PGF2α (10 µM) and IL-1β (10 ng/mL) for 16 h. (B) Translocation of NF-κB from the cytoplasm to the nucleus. HUtSMCs were pretreated with PDR97 for 30 min, followed by coincubation with PGF2α (10 µM) and IL-1β (10 ng/mL) for 30 min. (C) Analysis of NF-κB protein levels in the cytoplasmic and nuclear fractions. HUtSMCs were pretreated with PDR97 for 30 min, followed by coincubation with PGF2α (10 µM) and IL-1β (10 ng/mL) for 30 min. (D) Quantification of NF-κB protein levels. The relative expression level of NF-κB protein was normalized to the PGF2α- and IL-1β-treated control group. Data are presented as mean ± SEM. Statistical analysis was performed using one-way ANOVA, followed by a nonparametric test. *⁣*^*∗*^*p*  < 0.05, *⁣*^*∗∗*^*p*  < 0.01, *⁣*^*∗∗∗*^*p*  < 0.001, *⁣*^*∗∗∗∗*^*p*  < 0.0001.

PGs, including PGF2α, activate NF-κB signaling via binding to the FP receptor (PGF receptor) [26]. Once activated, NF-κB promotes the expression of inflammatory cytokines such as TNF-α and IL-1β, as well as COX-2, which further amplifies PGF2α synthesis, exacerbating inflammation and pain [27, 28]. Thus, regulating NF-κB activity represents a promising strategy for alleviating dysmenorrhea. To determine whether PDR97 inhibits NF-κB activation, we performed a luciferase reporter assay to assess NF-κB activity. As expected, NF-κB activity was markedly upregulated in response to PGF2α and IL-1β stimulation. However, treatment with PDR97 significantly suppressed this activation in a dose-dependent manner (*p*  < 0.01 at 25 μg/mL; *p*  < 0.001 at 50 and 100 μg/mL; Figure 7A). NF-κB activation typically involves its translocation from the cytoplasm to the nucleus in response to external stimuli, where it triggers inflammatory and immune responses [27]. To evaluate whether PDR97 interfered with this translocation process, we conducted an immunofluorescence assay. Interestingly, nuclear translocation of NF-κB was prominently observed following PGF2α and IL-1β treatment. However, PDR97 at 50 and 100 μg/mL effectively inhibited this translocation (Figure 7B). To quantify this effect, we further analyzed NF-κB protein expression levels. Consistent with our previous findings, PDR97 significantly reduced nuclear translocation of NF-κB (*p*  < 0.01 at 50 μg/mL; *p*  < 0.001 at 100 μg/mL; Figure 7C,D). These results indicate that PDR97 may alleviate dysmenorrhea by suppressing the NF-κB signaling pathway, thereby mitigating PGF2α-induced inflammation and pain.

## 4. Discussion

Excessive uterine contraction and ischemic vasoconstriction are primarily induced by increased levels of PGF2α and inflammatory mediators, which are key contributors to PD and significantly affect the quality of life in affected women [4, 29]. Currently, the most widely used therapeutic strategy for alleviating PD involves the administration of NSAIDs such as ibuprofen and naproxen, which are nonselective COX inhibitors [30, 31]. Psychological management techniques are sometimes incorporated into complementary approaches. In the present study, we evaluated the effects of *Aster spathulifolius* Maxim. extracts (PDR97) on uterine contractions and pain relief in a PD animal model. Additionally, we investigated the biological mechanisms in HUtSMCs. By elucidating these mechanisms, we propose PDR97 as a potential natural therapeutic agent that could serve as an alternative or complementary approach to NSAIDs, thereby offering plant-derived functional foods or pharmaceutical materials for the management of PD.

Previous studies have reported the efficacy of natural compounds for the management of PD. Wong et al. [21] demonstrated that the essential oil from *Salvia sclarea* L. alleviated uterine contractions in a PD animal model, while exhibiting antioxidant effects in uterine tissues. Similarly, Yang et al. [32] identified the therapeutic potential of peony pollen extract in modulating the COX-2/PGE2 pathway to improve PD symptoms. Our behavioral experiments further supported these findings, as PDR97 significantly reduced pain responses in an animal model of PD, indicating its potential analgesic effects. Histological analyses revealed that PDR97 normalized the contraction of the uterine horn and restored the length of the basal layer of the endometrium. Moreover, PDR97 effectively regulates COX-2 expression and exerts antioxidant effects, suggesting its protective role in the uterus by modulating inflammation and oxidative stress pathways.

NF-κB plays a crucial role in the pathogenesis of PD by regulating the expression of inflammatory cytokines and contraction-related proteins. Our study demonstrated that PDR97 significantly reduced NF-κB phosphorylation and nuclear translocation, leading to the suppression of contraction-related protein expression. These findings suggest that PDR97 mitigates excessive uterine contraction and inflammation through direct regulation of NF-κB signaling. Previous studies have reported that NF-κB activation in uterine smooth muscle cells enhances COX-2 expression, promotes PGF synthesis, and induces uterine contractions [28]. While NSAIDs act by directly inhibiting COX-2, PDR97 appears to exert broader antiinflammatory and anticontraction effects by targeting upstream NF-κB activation. Additionally, our study found that PDR97 significantly attenuated PGF2α- and IL-1β-induced ROS production. Excessive ROS generation exacerbates inflammation and uterine contractions, thereby aggravating PD symptoms. PDR97 may contribute to the overall suppression of NF-κB activity and inflammatory responses by reducing oxidative stress. Furthermore, PDR97 markedly decreased the expression of key inflammatory cytokines, including IL-1β, which plays a crucial role in sensitizing nociceptive pathways and amplifying pain perception. These findings supported the hypothesis that the antiinflammatory properties of PDR97 underlie its analgesic effects.

Our findings suggest that PDR97 has the potential to serve as a novel alternative or adjunct therapy for the management of PD. This could be particularly beneficial for patients who experience adverse effects of NSAIDs. Unlike conventional NSAIDs that primarily target COX-2, PDR97 exhibits a multifaceted mechanism of action by inhibiting NF-κB activation, reducing ROS levels, and modulating contraction-related proteins, thereby offering a comprehensive therapeutic effect. Additionally, given that NF-κB dysregulation is implicated in other gynecological disorders, such as endometriosis and uterine fibroids, PDR97 may have therapeutic potential beyond PD.

Despite these promising findings, this study has several limitations. First, although the effects of PDR97 were confirmed in a PD model, further research is needed to elucidate its interactions with other signaling pathways such as MAPK, PI3K/Akt, and COX-2. Second, isolation and identification of active compounds from PDR97 are necessary to gain deeper insights into their bioactive mechanisms. Third, clinical studies are required to validate the safety and efficacy of PDR97 in humans and to determine its optimal dosage and formulation for therapeutic applications.

## 5. Conclusions

In this study, we demonstrated that *Aster spathulifolius* Maxim. extract (PDR97) can alleviate pain, reduce inflammation, and suppress uterine contractions in a model of PD. Notably, its effects were found to be mediated through NF-κB inhibition and ROS reduction, highlighting its potential as a novel therapeutic agent for PD. Future studies are needed to explore the clinical applicability of PDR97 and evaluate its efficacy in other uterine-related disorders.

## Data Availability

The data that support the findings of this study are available from the corresponding author upon reasonable request.

## Supporting Information

Supporting Information Further supporting information is available online in the Supporting Information section. Supporting Information 1 Table S1. List of primary and secondary antibodies used in the immunoblotting and immunofluorescence.
