# Supplementary material for: Aster spathulifolius Maxim. Alleviates Primary Dysmenorrhea in a Mouse Model by Modulating Myometrial Contractions via NF-κB/COX-2 Pathway Inhibition
Source: Mediators Inflamm. 2025 Aug 12;2025:1654087. doi: 10.1155/mi/1654087 (PMC12364593; doi:10.1155/mi/1654087)
Supplement: Supporting Information — Further supporting information is available online in the Supporting Information section. Supporting Information 1 Table S1. List of primary and secondary antibodies used in the immunoblotting and immunofluorescence. [file 1654087.f1.docx]

**Mediators of Inflammation – Research Article – Supplementary material**

***Aster spathulifolius Maxim.* Alleviates Primary Dysmenorrhea in a Mouse Model by Modulating Myometrial Contractions via NF-κB/COX-2 Pathway Inhibition**

Min-Soo Kim^1,†^, Kang-In Lee^2,†^, Heung Joo Yuk^2^, Yousang Jo^2^, Hyungjun Kim^2^, and Ki-Sun Park^2,*^

^1^KM Convergence Research Division, Korea Institute of Oriental Medicine, Daejeon 34054, Republic of Korea.

^2^KM Science Research Division, Korea Institute of Oriental Medicine, Daejeon 34054, Republic of Korea.

^†^These authors contributed equally.

^*^ Corresponding author: kisunpark@kiom.re.kr

Tel.: +82-42-868-9662

**Table S1. List of primary and secondary antibodies used in the immunoblotting and immunofluorescence.**

| Antibody | Manufacturer | Catalog number | Dilution  (Primary) | Dilution  (Secondary) |
| --- | --- | --- | --- | --- |
| β-actin | Cell signaling, USA | 4967s | 1:7500 | 1:7500 |
| OTR | Alomone Labs, Israel | AVR-013 | 1:400 | 1:1000 |
| COX-2 | Santa Cruz, USA | SC-1745 | 1:500 | 1:1000 |
| p-ERK | Cell signaling, USA | 9101S | 1:1000 | 1:1000 |
| ERK | Cell signaling, USA | 9102S | 1:1000 | 1:1000 |
| p-MLC20 | Cell signaling, USA | 3675S | 1:1000 | 1:1000 |
| MLC20 | Abcam, USA | ab48003 | 1:500 | 1:1000 |
| p-NF-kB | Cell signaling, USA | 3033S | 1:500 **(IB)**  1:750 **(IF)** | 1:1000 **(IB)**  1:200 **(IF)** |
| NF-kB | Cell signaling, USA | 8482S | 1:1000 | 1:1000 |
| iNOS | Abcam, USA | ab15323 | 1:1000 | 1:1000 |
| IL-6 | Invitrogen, USA | P620 | 1:500 | 1:1000 |
| TNF-α | Santa Cruz, USA | SC-52746 | 1:500 | 1:1000 |
| GAPDH | Cell signaling, USA | 2118 | 1:1000 | 1:1000 |
| Lamin A/C | Cell signaling, USA | 2032 | 1:1000 | 1:1000 |
| Anti-rabbit IgG, HRP-linked | Cell signaling, USA | 7074S | - | - |
| Anti-mouse IgG, HRP-linked | Cell signaling, USA | 7076S | - | - |
| Donkey anti-goat IgG-HRP | Santa Cruz, USA | SC-2020 | - | - |
| Alexa Fluor 488 donkey anti-rabbit IgG | Invitrogen, USA | A21206 | - | - |

**Abbreviation: IB,** Immunoblotting**; IF,** Immunofluorescence
